# Finnish gelsolin amyloidosis causes significant disease burden but does not affect survival: FIN-GAR phase II study

**DOI:** 10.1186/s13023-020-1300-5

**Published:** 2020-01-17

**Authors:** Eeva-Kaisa Schmidt, Tuuli Mustonen, Sari Kiuru-Enari, Tero T. Kivelä, Sari Atula

**Affiliations:** 1 Clinical Neurosciences, Neurology, University of Helsinki and Helsinki University Hospital, HYKS, Tornisairaala, Neupkl, Haartmaninkatu 4, 00029 HUS Helsinki, Finland; 2 Department of Ophthalmology, University of Helsinki and Helsinki University Hospital, Helsinki, Finland

**Keywords:** Gelsolin, Amyloidosis, AGel, Hereditary amyloidosis, Meretoja syndrome, Natural history, Lifespan, Relative survival

## Abstract

**Background:**

Hereditary gelsolin (AGel) amyloidosis is an autosomal dominantly inherited systemic amyloidosis that manifests with the characteristic triad of progressive ophthalmological, neurological and dermatological signs and symptoms. The National Finnish Gelsolin Amyloidosis Registry (FIN-GAR) was founded in 2013 to collect clinical data on patients with AGel amyloidosis, including altogether approximately one third of the Finnish patients. We aim to deepen knowledge on the disease burden and life span of the patients using data from the updated FIN-GAR registry. We sent an updated questionnaire concerning the symptoms and signs, symptomatic treatments and subjective perception on disease progression to 240 members of the Finnish Amyloidosis Association (SAMY). We analyzed the lifespan of 478 patients using the relative survival (RS) framework.

**Results:**

The updated FIN-GAR registry includes 261 patients. Symptoms and signs corresponding to the classical triad of ophthalmological (dry eyes in 93%; corneal lattice amyloidosis in 89%), neurological (numbness, tingling and other paresthesias in 75%; facial paresis in 67%), and dermatological (drooping eyelids in 86%; cutis laxa in 84%) manifestations were highly prevalent. Cardiac arrhythmias were reported by 15% of the patients and 5% had a cardiac pacemaker installed. Proteinuria was reported by 13% and renal failure by 5% of the patients. A total of 65% of the patients had undergone a skin or soft tissue surgery, 26% carpal tunnel surgery and 24% at least unilateral cataract surgery. As regards life span, relative survival estimates exceeded 1 for males and females until the age group of 70–74 years, for which it was 0.96.

**Conclusions:**

AGel amyloidosis causes a wide variety of ophthalmological, neurological, cutaneous, and oral symptoms that together with repeated surgeries cause a clinically significant disease burden. Severe renal and cardiac manifestations are rare as compared to other systemic amyloidoses, explaining in part the finding that AGel amyloidosis does not shorten the life span of the patients at least for the first 75 years.

## Background

Hereditary gelsolin amyloidosis (AGel amyloidosis; OMIM#105120), also called Meretoja syndrome according to the Finnish ophthalmologist Jouko Meretoja who first described it in 1969 [1], is an autosomal dominantly inherited systemic amyloidosis. The most common clinical signs in patients with AGel amyloidosis are progressive ophthalmological (corneal lattice amyloidosis), neurological (cranial and peripheral neuropathy), and dermatological (cutis laxa) signs, manifesting typically in their forties or fifties [2, 3]. Also other internal organ, especially renal [4–6], and cardiac [4, 5, 7–10], manifestations have been reported. The symptoms of AGel amyloidosis are suggested to be based on the deposition of gelsolin-based amyloid (AGel) fibrils and pre-amyloid oligomers, originating from misfolded gelsolin fragments and accumulating in multiple organs and tissues [2, 11]. So far it is not known why the manifestations of the disease vary remarkably between the patients [2].

Gelsolin is a calcium-activated, actin-modulating protein that has a role in multiple biological processes, and is present both in cytosolic and secretory forms in most cells [12–14]. AGel amyloidosis is caused either by the point mutation c.640G > A in the gelsolin gene on chromosome 9 at q33.2 [15, 16] or, less frequently, by the mutation c.640G > T at the same locus [2, 8, 17–23]. Two novel gelsolin gene variants (c.633C > A, c.580G > A) causing renal amyloidosis have recently been reported in the US [24, 25]. The penetrance of the c.640G > A pathogenic variant is 100%. The prevalence of this mutation in Finland seems to be higher than anywhere else but individual patients and kindreds have also been reported in several other countries [2]. Estimates of the number of patients in Finland vary from 600 to 1000 [2, 3].

Meretoja himself began to chart the natural course of AGel amyloidosis [1]. Since his time, the prevalence and progression of its various signs and symptoms have been reported in several smaller cohorts and case reports. Our research group founded the National Finnish Gelsolin Amyloidosis Registry (FIN-GAR) in 2013 in order to increase the understanding on the natural course of this rare amyloidosis. Altogether 227 patients (211 living, 16 deceased) were entered in the registry in 2013–2014, estimated to cover approximately one third of the Finnish patients [3]. Our study showed that the patients become symptomatic at the mean age of 39 years and that ophthalmologic symptoms were the first to appear. The high prevalence of the characteristic triad of ocular, neurological and cutaneous symptoms was demonstrated [3]. However, open questions remained concerning the less well known of the anticipated symptoms, the symptomatic treatments, and disease progression.

Meretoja suggested in 1973 that the mortality of AGel amyloidosis patients might be somewhat increased [26]. In a previous study by our research group, however, based on death certificates of 231 patients who died in 1980–2014, the lifespan was shown to be comparable to that of the general Finnish population [27]. That study was based exclusively on data from deceased patients, which might have caused bias, and thus the result must be confirmed in a representative patient group including both the living and the deceased patients.

In our present study (FIN-GAR phase II), the data in the FIN-GAR patient registry were updated and 29 new patients were entered into the registry, increasing the total number of the patients to 261. More comprehensive overview of their various signs and symptoms, symptomatic treatments, and disease progression was obtained by introducing a more detailed survey. In addition, our study increases knowledge on the survival of AGel amyloidosis patients. We compared the lifespan of 478 living and deceased patients to that of the general Finnish population, utilizing relative survival analysis. To our knowledge, this is the most comprehensive study on the natural history of AGel amyloidosis.

## Results

The FIN-GAR phase II questionnaire was returned by 129 patients, which corresponds to 54% of SAMY members. Of these 129 patients, 100 (47% of the surviving patients) had taken part in FIN-GAR phase I in 2013–2014, and 29 patients were new. As a result, FIN-GAR phase II registry includes 261 patients, of whom 183 (70%) are females and 78 (30%) males. Their mean age is 62.7 years (range, 26 to 85). Genetic testing to confirm the AGel c.640G > A variant had been done for 138 (53%) patients, one of whom is a homozygote. The mean follow-up time for the patients included in both phases was 4.9 years (range, 4.1–5.6). Baseline information regarding patients in the FIN-GAR phase II registry is presented in Table 1. The symptoms, their frequency and the median age at onset are presented in Table 2, and the percentage of patients receiving specific symptomatic treatments is presented in Table 3.

**Table 1:** Baseline information on the patients with AGel amyloidosis in the FIN-GAR phase II registry

Number of patients	261
Gender distribution	183 (70%) female, 78 (30%) male
Mean age of the patients	62.7 years (range 26.3–85.8 years)
Median age of the patients	64.7 years (interquartile range 54.9–71.2 years)
Mean age at diagnosis of AGel amyloidosis	40.7 years (range 10.4–68.7 years)
Genetic testing done	138 (53%)
Follow-up time after FIN-GAR phase I	4.9 years (range 4.1–5.6 years)

**Table 2:** The symptoms and signs of the patients with AGel amyloidosis in the FIN-GAR phase II registry by order of frequency and the age at the onset of symptoms

	Frequency (95% CI)	n	Total	Median age (y)	Interquartile range (y)
Eyes					
Dry eyes	93% (90–96)	242	261	45	37–51
Corneal lattice amyloidosis	89% (85–92)	231	261	42	34–51
Photophobia	78% (71–86)	101	129	49	39–61
Impaired vision	78% (73–83)	204	261	51	41–61
Corneal ulcer	50% (44–56)	131	261	49	40–60
Cataract	47% (41–53)	123	261	62	57–68
Tearing	26% (19–34)	34	129	56	46–62
Glaucoma	23% (18–28)	60	261	61	51–66
Skin and soft tissues					
Drooping eye lids	86% (82–90)	225	261	50	42–57
Cutis laxa	84% (79–88)	219	261	50	42–57
Dry skin	79% (74–84)	206	261	40	17–54
Bruising	69% (63–75)	180	261	39	26–47
Wounds, skin vulnerability	64% (55–72)	82	129	34	15–49
Itchy skin	33% (27–38)	85	261	49	29–60
Varicose veins	30% (22–38)	39	129	36	29–47
Haemorrhoids	29% (21–37)	38	129	42	30–52
Hernia of any type	11% (8–15)	30	261	49	41–63
Prolapse of any type	8% (5–11)	21	261	41	32–53
Neurology					
Numbness, tingling and other paresthesias	75% (69–80)	195	261	56	44–63
Facial paresis	67% (62–73)	176	261	50	44–59
Myokymia	54% (48–60)	140	261	48	41–58
Carpal tunnel syndrome	43% (36–49)	111	261	50	37–61
Balance impairement	39% (33–45)	103	261	64	55–70
Dysarthria	31% (26–37)	81	261	61	54–68
Kidneys					
Proteinuria	13% (9–18)	35	261	54	35–59
Renal failure	5% (3–8)	14	261	63	61–71
Heart					
Arrhytmias	16% (9–22)	20	129	52	30–66
Ischaemic heart disease	9% (4–14)	12	129	54	43–73
Cardiomyopathy	5% (2–8)	13	261	58	43–65
Valvulopathy	5% (3–8)	14	261	61	35–73
Atrial fibrillation	5% (1–8)	6	129	54	N/A
Cardiac failure	5% (1–8)	6	129	61	42–74
Oral health					
Dry mouth	43% (36–49)	111	261	55	41–63
Caries	43% (34–51)	55	129	24	6–39
Gingivitis	19% (12–26)	25	129	54	32–59
Other symptoms					
Pain	38% (32–44)	99	261		
Oedema	28% (22–33)	72	261		
Malignancy	15% (9–21)	19	129		
Depression	13% (7–19)	17	129		
Impaired hearing	43% (37–49)	112	261		
Hypothyreosis	10% (6–13)	25	261		
Sleep apnoea	6% (3–9)	15	261

**Table 3:** Patients with AGel amyloidosis in the FIN-GAR phase II registry with specific symptomatic treatments

Symptomatic treatment and follow	Frequency (%)	n	Total
Moisturizing skin creams	85	110	129
Skin and soft tissue surgery	65	170	261
Artificial tears	46	121	261
Carpal tunnel syndrome surgery	26	68	261
Cataract surgery	24	62	261
Hearing aid or cochlear implant	9	23	261
Pacemaker	5	12	261
Kidney transplantation	2	5	261
Dialysis	1	3	261

### Ophthalmological manifestations

Corneal lattice amyloidosis (previously incorrectly called lattice corneal dystrophy, type II) is the first ophthalmological sign to appear (median age at diagnosis, 41 years) and 231 (89%) patients reported to suffer from it. Dry eyes is an even more common problem, affecting 242 (93%) patients (median age, 45 years). Photophobia and tearing were inquired separately only in the phase II questionnaire and the results are based on those 129 patients. Photophobia was reported by 101 (78%) patients (median age, 49 years) and tearing by 34 (26%) patients (median age, 56 years). Cataract was reported by 123 (47%) patients (median age, 62 years) and glaucoma by 60 (23%) patients (median age, 61 years). Of patients included in phase I and II, 95 (74%) told that their ophthalmological symptoms had progressed in the past 5 years.

**Fig. 1:**
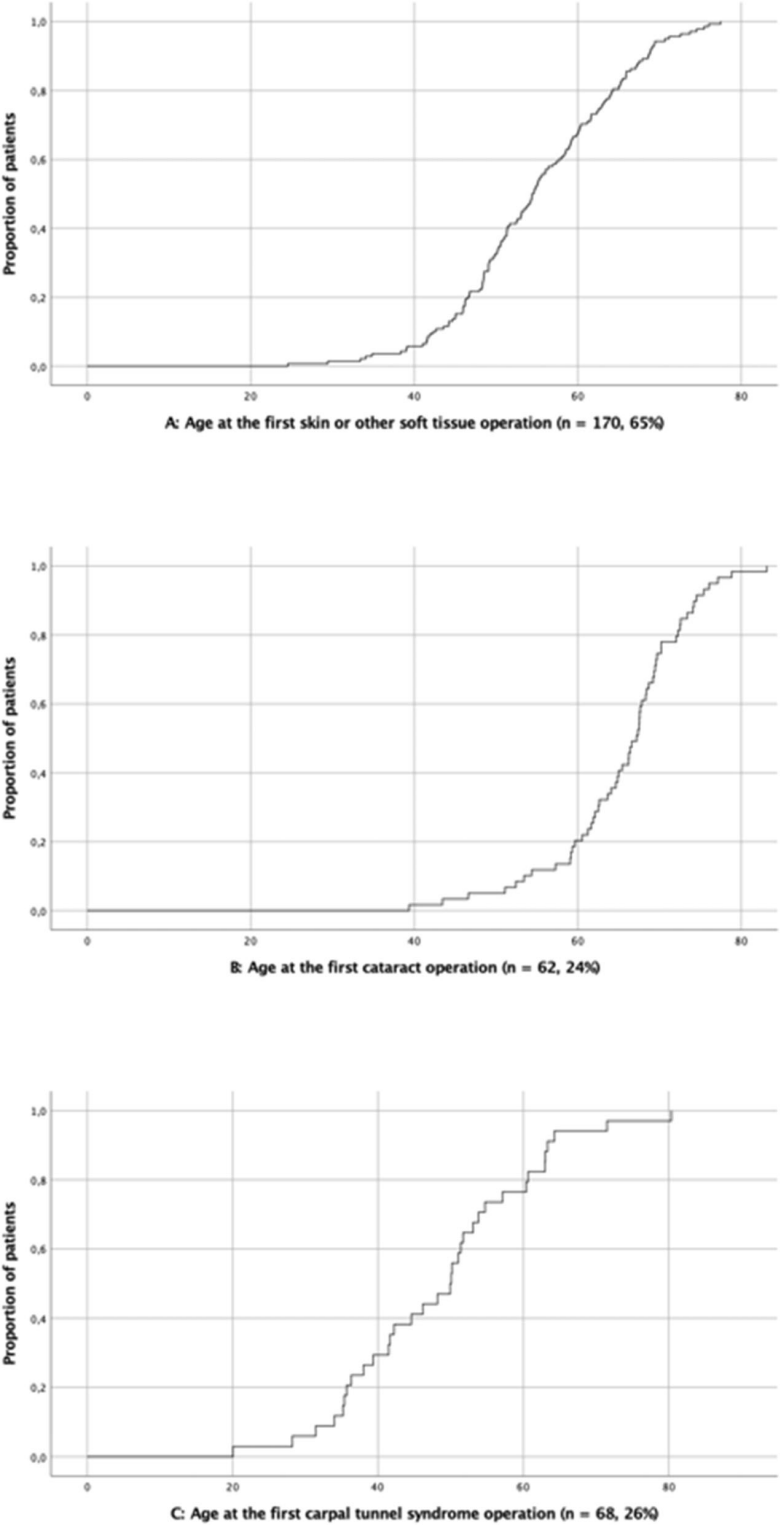
The cumulative frequency of selected surgeries in 261 patients with AGel amyloidosis in the FIN-GAR phase II registry. **a**: Skin or other soft tissue surgeries (*n* = 170, 65%). **b**: Cataract surgeries (*n* = 62, 24%). **c**: Carpal tunnel syndrome surgeries (*n* = 68, 26%)

Artificial tears were used by 121, approximately one half (46%) of the patients, and 197 (75%) visited their ophthalmologist regularly. At least unilateral cataract surgery was performed for 62 (24%) patients. The median age at the time of the first cataract surgery was 67 years. The cumulative frequency of selected surgeries is presented in Fig. 1.

### Cutaneous and soft tissue manifestations

The most prevalent cutaneous symptom was drooping eyelids, caused by cutis laxa and aggravated by facial nerve paresis, reported by 225 (86%) patients (median age at onset, 50 years). Cutis laxa, abnormal general loosening of the skin, was reported by 219 (84%) patients (median age, 50 years), dry skin by 206 (79%) patients (median age, 40 years), and itchiness of the skin by 85 (33%) patients (median age, 49 years). Consequently, 110 (85%) patients used regularly moisturizing skin creams. The skin appears to be vulnerable to injury: 180 (69%) patients (median age, 39 years) suffered from tendency to bruising and 82 (64%) patients (median age, 34 years) reported getting wounds easily.

Manifestations of AGel amyloidosis may also include pathological changes in soft tissues other than the skin. Varicose veins were reported by 39 (30%) patients (median age at onset, 36 years), haemorrhoids by 38 (29%) patients (median age, 42 years), and hernias of different types by 30 (11%) patients (median age, 49 years), whereas 21 (8%) patients reported at least one prolapse of any type (median age, 41 years). Of patients who took part in both phases, 95 (72%) had the perception that their skin and other soft tissue-related symptoms had progressed during the follow-up.

**Fig. 2:**
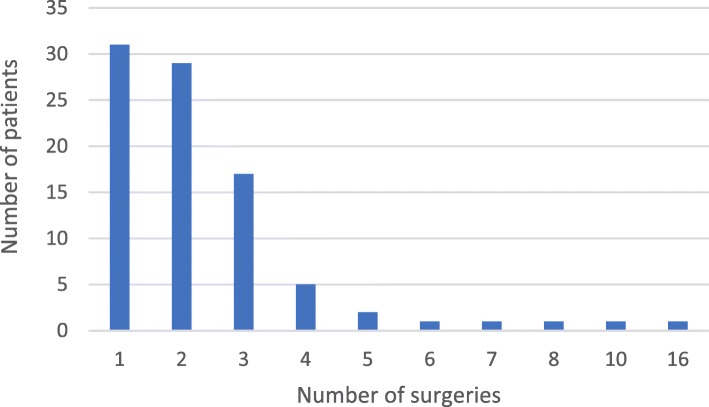
Number of skin surgeries in 89 patients with AGel amyloidosis in the FIN-GAR phase II registry, including eyelid and other facial surgeries, hernia and prolapse surgeries, and varicose vein surgeries

Skin or other soft tissue surgeries had been performed in 261 (65%) patients. On average, patients had gone through 2.4 such surgeries (range, 1–16; Fig. 2) and the median age at the time of the first surgery in 129 patients was 53 years (interquartile range, 48–72 years).

### Neurological manifestations

The most prevalent neurological symptoms were numbness, tingling and other paresthesias, reported by 195 (75%) patients (median age at onset, 56 years). Facial paresis in 176 (67%) patients become symptomatic at the median age of 50 years. Other frequent manifestations were myokymias (54%; median age, 48 years), carpal tunnel syndrome (43%, 50 years), balance impairment (39%, 64 years) and dysarthria (31%, 61 years). Patients with dysarthria reported especially difficulty in articulating clearly outdoors in cold weather. Approximately half (53%) of the patients participating in both phases stated that their neurological symptoms had progressed during the follow-up. Of 111 (43%) patients with a carpal tunnel syndrome diagnosed, 68 (61%) underwent surgery at a median age of 50 years (Fig. 1).

### Cardiovascular manifestations

In the updated questionnaire, we asked spesifically about cardiac arrhythmias other than atrial fibrillation, whereas in the previous questionnaire there was only a general question regarding arrhythmias. Of patients who returned the updated questionnaire, 20 (15%) reported cardiac arrhythmias and 6 (5%) atrial fibrillation, another 6 (5%) suffered from cardiac failure, and 12 (9%) had an ischaemic heart disease. Cardiomyopathy was reported by 13 (5%) patients. Pacemaker was installed for 12 patients (5%), and 8 (6%) patients reported a history of any kind of cardiac surgery of which 4 were bypass surgeries or angioplastias. The majority (61%) of the patients were not aware of any cardiovascular signs or symptoms, excluding hypertension and hypercholesterolemia. However, 12 (9%) patients reported that they had experienced progression in their cardiac symptoms during the follow-up.

### Renal manifestations

Proteinuria had been detected in 35 (13%) patients (median age at onset, 54 years). Renal failure was reported by 14 (5%) patients and two patients (2%) used medications for renal disease. Moreover, 3 (1%) patients received or had previously received dialysis treatment, and kidney transplantation had been performed to 5 (2%) patients. Conversely, 111 (86%) patients who returned the updated questionnaire reported no kidney-related signs or symptoms, and only 6 (5%) had the perception that their renal symptoms had progressed during the follow-up.

### Other manifestations

Regarding oral health, 111 patients (43%) reported dry mouth, 55 (43%) caries, and 25 (19%) gingivitis. One fifth of the patients (19%) reported no oral health issues, but approximately one third (35%) reported worsening oral health.

Patients also reported other symptoms possibly linked to AGel amyloidosis based on previous studies. Impaired hearing was reported by 112 (43%) patients and 23 (9%) patients had a hearing aid or cochlear implant. Oedema as an unspecific symptom, possibly in some patients related to renal problems, was reported by 72 (28%) patients. Sleep apnoea was reported by 15 (6%), hypothyroidism by 25 (10%), different kinds of pain by 99 (38%), cancer by 19 (15%), and depression by 17 (13%) patients.

### Retirement and relative survival

Of the male and female patients, 42 (54%) and 99 (54%) had retired at mean age of 57.9 and 59.1 years, respectively.

**Fig. 3:**
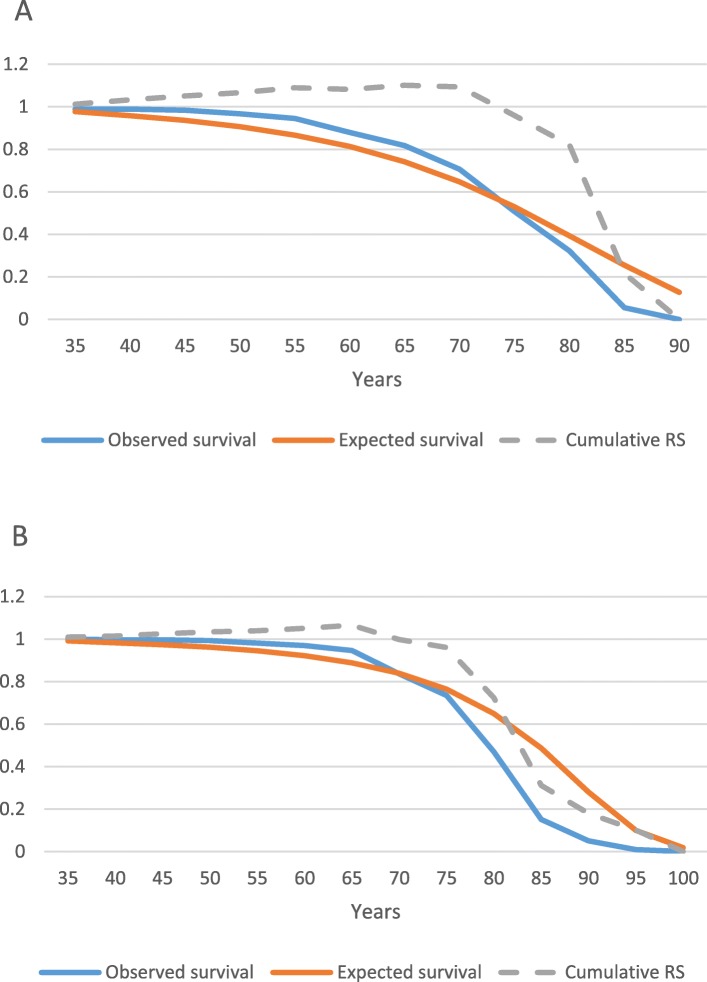
Relative survival of **a**) males and **b**) females with AGel amyloidosis in the FIN-GAR phase II registry and a previous study on survival. RS = relative survival [27]

We compared the lifespan of the patients with that of the general Finnish population using the relative survival framework (Table 4, Fig. 3). Relative survival was comparable to that of the general population at least until the age of 75 years: in the age group of 70–74 years old, i.e. after 30 to 40 years after the first symptoms, the relative survival was 0.96 for both male and female patients. After the age of 75, less than 100 patients remained in the analysis and the observed survival was thereafter lower than the expected survival for both genders.

**Table 4:** Relative survival for 188 male and 290 female patients with AGel amyloidosis in the FIN-GAR phase II registry and a previous study on survival [27]

Age group	n in the beginning	Observed survival	Expected survival	Cumulative relative survival
Male				
30–34	188	0.9893	0.9777	1.0119
35–39	185	0.9893	0.9577	1.0330
40–44	183	0.9839	0.9360	1.0511
45–49	180	0.9674	0.9065	1.0671
50–54	174	0.9448	0.8669	1.0899
55–59	165	0.8800	0.8136	1.0817
60–64	145	0.8172	0.7415	1.1021
65–69	125	0.7073	0.6468	1.0936
70–74	97	0.5085	0.5300	0.9594
75–79	62	0.3221	0.3927	0.8200
80–84	36	0.0552	0.2550	0.2165
85–89	5	0.0000	0.1274	0.0000
Female				
30–34	290	1.0000	0.9907	1.0094
35–39	287	0.9965	0.9820	1.0147
40–44	284	0.9965	0.9728	1.0244
45–49	280	0.9929	0.9610	1.0332
50–54	267	0.9813	0.9448	1.0387
55–59	246	0.9688	0.9220	1.0507
60–64	222	0.9459	0.8883	1.0648
65–69	195	0.8370	0.8386	0.9982
70–74	149	0.7339	0.7641	0.9605
75–79	110	0.4703	0.6494	0.7241
80–84	59	0.1513	0.4863	0.3112
85–89	17	0.0504	0.2809	0.1796
90–94	5	0.0101	0.0994	0.1015
95–99	1	0.0000	0.0182	0.0000

## Discussion

Our registry study, FIN-GAR phase II, included 261 Finnish AGel amyloidosis patients of whom 29 were new. The registry is estimated to represent 25–40% of Finnish AGel patients, which we consider to be a reasonably large sample of all patients suffering from this rare disease. Our study describes the typical course of the disease and shows that symptoms and signs corresponding to the classical triad of ophthalmological (dry eyes in 93%; corneal lattice amyloidosis in 89%), neurologic (numbness, tingling and other paresthesias in 75%; facial paresis in 67%), and cutaneous (drooping eyelids in 86%; cutis laxa in 84%) manifestations are highly prevalent. Our study increases knowledge on the diversity of symptoms by showing that, in addition to previously known ones, a significant proportion of patients suffer from symptoms such as photophobia and a hearing deficit.

The prevalence of various ophthalmological manifestations was shown to be high in AGel amyloidosis patients but comparison data on prevalence of dry eyes, photophobia and tearing in the general population is not available. In a population based survey on the prevalence of major eye diseases in Finland [28], partly based on self-reported data similarly to this study, the prevalence of cataract and glaucoma for persons aged 30 years and older was reported to be 10 and 5%, and for persons aged 65 years and older 34 and 13%, respectively. In FIN-GAR registry patients with median age of 62.7 years the prevalence of cataract was 47% and that of glaucoma 23%. Cataract and glaucoma have been associated to AGel amyloidosis [4, 6, 29–35], but this high prevalence in patients was new information received through this study.

Understanding cardiac manifestations in AGel amyloidosis has been mainly based on smaller patient series and case reports that have reported conduction defects, such as atrioventricular blocks, sinus bradycardia, and sick sinus syndrome [4, 7–10]. In a series of 30 patients, signs of amyloid cardiopathy were rare both at clinical and in radiologic, echocardiographic and electrocardiographic examinations in middle-aged patients [36]. Valvulopathies have been observed in occasional patients [5, 7, 8]. Finally, only one patient with heart transplantation has been reported [37]. Our study confirms the previous assumption that severe clinical cardiac manifestations in AGel amyloidosis are rather rare, especially when compared with other systemic amyloidoses, such as AL and transthyretin (ATTR) amyloidosis. This is interesting, knowing that all investigated patients in our recent autopsy study of 25 patients had amyloid deposits in both myocardium and in cardiac blood vessels [38]. However, the study revealed that 5% of the patients have a pacemaker. It is more than in the general population: in year 2013 in Finland a total of 1020 new pacemakers per million inhabitants were installed [39], and the total prevalence of pacemakers in the population of the Helsinki University Hospital area is approximately 1% [40]. Relatively low frequency of severe cardiac manifestations likely contributes to the reasonably normal life span of AGel patients. However, notable individual variation in the phenotype means that, in rare cases, cardiac manifestations can be significant.

Similarly, the assumption has been that renal manifestation of AGel amyloidosis are rather rare and mild in heterozygous patients [4–6]. However, some patients have developed even a nephrotic syndrome [26, 35, 41], requiring dialysis or kidney transplantation. On the other hand, homozygous patients have severe renal manifestations and they may suffer from nephrotic syndrome already in their early twenties, progressing to renal failure, dialysis treatment, and death [26, 42, 43]. The two recently reported gelsolin gene variants c.580G > A and c.633C > A seem to exclusively cause renal amyloidosis [24, 25]. Before the FIN-GAR registry, the true frequency of renal signs and symptoms was unknown. Our study confirms that severe renal manifestations are rare in AGel amyloidosis.

Hyposalivation and altered salivary composition have been suggested to increase the risk of caries and oral candidiasis in patients with AGel amyloidosis [44]. In our study, over 40% of the patients reported that they suffered from a dry mouth, and equally many from a tendency to caries, indicating that oral health care is an important part of symptomatic treatment of the patients.

The high frequency of skin and soft tissue surgeries is also an interesting observation. As many as 65% of the patients had been operated on at least once, and repeated skin or soft tissue surgeries had been completed for 45% of them. Tissue laxity was not limited to the skin, because patients reported also varicose veins, haemorrhoids, hernias, and prolapses, frequently requiring surgical treatment. However, the prevalence of varicose veins does not seem to be higher than in the general population [45]. An earlier study suggested that repeated plastic surgery is needed because its effects, although satisfactory, are only short-term [46]. Facial surgeries are likely due to the progressive changes in the structure of the skin. An increasing amount of amyloid in the skin is found with advancing age, and amyloid spreads even to the deeper dermis and subcutaneous adipose tissue [47]. In addition, the characteristic facial nerve palsy that begins from the frontal branch of the nerve hastens the visible facial changes. In a series of 35 operated patients, none had normal function of the frontal branch of the facial nerve, and weakness in the buccal branch was seen in 40% [46].

The patients (42 males and 99 females) had retired between 1968 and 2019. Statistical data on the age of retirement in Finland is available only for years 1996–2018. During that period, the mean age of retirement varied from 56.6 to 60.9 years for males and from 57.8 to 60.7 years for females [48]. Males and females with AGel amyloidosis retired at the mean age of 57.9 years and 59.1 years, respectively. AGel amyloidosis does not seem to have a major influence on the age of retirement.

The lifespan of the patients was modeled using the relative survival framework, and the result of a previous study [27] was confirmed: the lifespan was not shorter than that of general Finnish population. The explanation why the observed survival of the patients was lower than the expected one in the oldest age groups is likely the small number of patients in these cohorts, causing bias. AGel amyloidosis seems to be an exception among other systemic amyloidosis in respect to the life span. Of other hereditary systemic amyloidoses, ATTR amyloidosis leads to death on average in 10 years after the disease onset [49], cystatine (ACys) amyloidosis before the age of 40 years [50], fibrinogen (AFib) amyloidosis within a median of 15.2 years after disease manifestation [51], and in lysozyme (ALys) amyloidosis the survival varies remarkably from 1 to 20 years after disease onset [52]. Especially the relative rarity of severe cardiac and renal manifestations likely contribute to this finding. The preliminary finding of our previous study [27] that the frequency of cancer as a cause of death in patients with AGel amyloidosis is lower than in the general population may also have an influence on their survival. It is interesting that although the disease burden is significant, the lifespan is comparable to that of the general population, at least until the age of 75 years, and the patients do not retire earlier than the general population.

Our study has several limitations. Of the surviving patients in the FIN-GAR phase I registry, 47% responded in the phase II questionnaire, and the results are partly based on the information reported already in our previous study [3]. The data were self-reported and collected through a patient survey, and complemented by telephone interviews in the phase I study, and not all the reported signs and symptoms were verified by a physician, which may artificially increase their prevalence. However, we consider it unlikely that patients would report certain signs like cataract or glaucoma without having a diagnoses made by an ophthalmologist. As the symptoms begin gradually, over many years, and many patients have a spectrum of different symptoms, memorizing the exact starting point of each symptom is subject to recall error. For this reason, the given years of onset must be taken as rough estimates in most cases. Secondly, patients were recruited through a disease-specific patient organisation. Patients registered as members might differ from those who declined such activities, as might patients who returned and did not return the phase II questionnaire either because they had less health problems or were more debilitated. Likewise, the gender distribution in our study was uneven, which may be due to the fact that female patients in general are more likely to answer questionnaires. On the other hand, our data represent an estimated 25–40% of all Finnish AGel amyloidosis patients, which provides reasonable assurance of representativeness.

## Conclusions

We have presented here the most up to date and comprehensive cross-sectional study on AGel amyloidosis. The results reported in FIN-GAR phase I study were confirmed and data on several new symptoms and signs, use of symptomatic treatment, and performed surgeries add to the knowledge on the course and the burden of AGel amyloidosis. Our study emphasizes the clinical significance of the classical triad of ophthalmologic, neurologic and cutaneuous symptoms and signs, and confirms the earlier hypothesis that cardiac and renal manifestations are not very common in this amyloidosis. However, for yet unknown reasons and possibly related to the variability in the phenotype, patients rarely may develop severe cardiac or renal amyloidosis, leading to organ transplantation or even death.

## Methods

Between September 2013 and June 2014 altogether 227 patients were entered into the FIN-GAR registry, and five more patients were added later in 2014–2017. In phase I, we interviewed most of the patients (87%) on telephone to complement the data given in the questionnaire. Full update (phase II) commenced in September 2018, 5 years after founding the registry.

An updated questionnaire, including a larger scale of known and anticipated symptoms and signs of AGel amyloidosis and their time of onset, was created. Because new patients were recruited, baseline information regarding primary symptoms, details of diagnosis and family history were inquired, similarly to the phase I questionnaire. In addition, the new questionnaire inqueried about possible symptomatic treatments that were either self-administered or provided by the health care system. Finally, the patients were asked about their subjective perception of the progression of their symptoms in different organs since the previous questionnaire, approximately 5 years earlier.

The Finnish Amyloidosis Association (SAMY; Suomen amyloidoosiyhdistys ry), sent the questionnaire to their 240 members. The pathognomonic clinical manifestations of AGel amyloidosis consist of the triad of corneal lattice amyloidosis, peripheral facial paresis and cutis laxa. This triad is not seen in any other known disorder [2], and for this reason, genetic testing is not required for diagnosis, especially if there are known cases of AGel amyloidosis in the family. Data received from patients who had participated in the FIN-GAR phase I were updated in the registry, and new patients, not previously entered in the registry, were included.

**Fig. 4:**
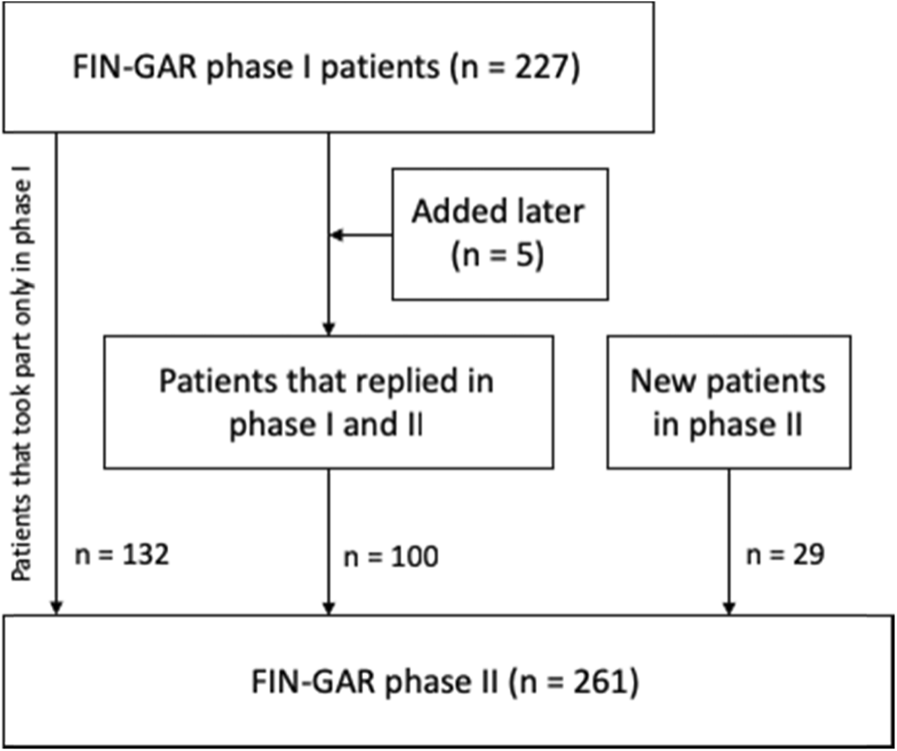
Of the patients that were included in the FIN-GAR phase I registry, 100 responded in the phase II questionnaire and their data was updated. In addition, 29 new patients were included in the registry. In the FIN-GAR phase II registry the data of 132 patients was obtained from the FIN-GAR phase I registry

Because several new questions were added to the FIN-GAR phase II questionnaire, part of the endpoints could be analysed only for those 129 patients who returned that questionnaire. The analysed patient group is indicated in Table 2 by each symptom and sign. Regarding the endpoints for which data were available also from the FIN-GAR phase I study, the registry data were updated and the analyses were done for the complete registry of 261 patients. The patient inclusion process is presented in Fig. 4. The frequencies and the median age at the onset of the symptoms/signs were calculated. Distributions of continuous variables were analysed and tested for normality. The cumulative frequency of selected surgeries was plotted according to the age of the patient at the time of the surgery. These figures were constructed based on data from the patients who had the symptom of interest. IBM SPSS version 25 (SPSS, Inc., an IBM company, New York, NY) was used for the analyses.

The lifespan of AGel amyloidosis patients was calculated based on survival data of 478 living and deceased patients. First, we extracted survival data from 272 death certificates that we collected for a previous study [27]. Then, survival data of 208 living patients was added from the FIN-GAR phase I patient registry; late patients of FIN-GAR phase I registry were already included in the data extracted from the death certificates. After data quality control we removed two subjects from the data set prior to the analysis. The lifespan was analysed using the relative survival framework, based on Ederer II estimator [53]. Relative survival is defined as the ratio of the all-cause survival of the patients to the all-cause survival that would be expected [54]. If relative survival is 1, the patient group survives equally in comparison to the background population, in this case general age, gender and calendar year matched Finnish population. If the disease of interest is rare, any difference in survival can be assumed to be due to that disease. Thus, the relative survival framework does not need information about the cause of death. Information concerning the general Finnish population was obtained from Statistics Finland. For the purpose of running the analysis, disease onset was set to the age of 30 years, and relative survival was estimated in five year time intervals using Stata (version 13, StataCorp, College Station, TX).

## Data Availability

The datasets used and/or analysed during the current study are available from the corresponding author on reasonable request.

## Abbreviations

ACys: Cystatine amyloid
AFib: Fibrinogen amyloid
AGel: Gelsolin amyloid
AL: Light-chain amyloidosis
ALys: Lysozyme amyloid
ATTR: Transthyterin amyloid
FIN-GAR: The National Finnish Gelsolin Amyloidosis Registry
RS: Relative survival
SAMY: Finnish Amyloidosis Association (Suomen amyloidoosiyhdistys ry)
